# Severe case of bullous pemphigoid associated with nivolumab responsive to combination therapy with dupilumab and omalizumab

**DOI:** 10.1016/j.jdcr.2024.12.014

**Published:** 2024-12-26

**Authors:** Liana Victory, Gregg Murray, Claire Quigley, Stephanie Bowe, Cliona Feighery, Ann Fortune, Ian McDonald

**Affiliations:** a Department of Dermatology, St George Hospital, Sydney, Australia; b Department of Dermatology, Mater Matericordiae University Hospital, Dublin, Ireland; c Department of Dermatology, Our Lady of Lourdes Hospital, Drogheda, Ireland; d Department of Haematology, Mater Misericordiae University Hospital, Dublin, Ireland

**Keywords:** biologics, bullous pemphigoid, dupilumab, immunobullous, omalizumab

## Introduction

Bullous pemphigoid (BP) secondary to immunotherapy has presented a new cohort of patients with many unique therapeutic challenges. A greater understanding of the pathophysiology of BP has led to the off-license use of newer targeted biologic therapies. Dupilumab and omalizumab are emerging as effective, targeted treatment options, particularly in cases recalcitrant to standard treatments.^1^, ^2^, ^3^, ^4^

Dupilumab is a fully human IgG4 monoclonal antibody acting as an antagonist against interleukin (IL)-4 receptor α. This results in the downregulation of TH2 cytokines IL-4 and IL-13, which are increased in the sera and blister fluid of patients with BP. It also downregulates immunoglobulin (Ig) E secretion and eosinophil activity, also thought to play a role in the pathogenicity of BP. Omalizumab is an anti-IgE monoclonal antibody and hence inhibits the activation of mast cells that are increased in lesions of BP.

## Case report

We report a severe case of recalcitrant BP secondary to nivolumab treated successively with omalizumab and dupilumab in combination. A 43-year-old female patient was referred to the dermatology department with a 2-week history of a pruritic blistering rash. She had previously been diagnosed with treatment-resistant Hodgkin’s lymphoma. She had failed multiple lines of chemotherapy and had subsequently been commenced on nivolumab, completing 14 cycles in August 2021. Her medical history also included hypothyroidism, obesity, epilepsy, and learning difficulty. Long-term medications included levothyroxine, carbamazepine, famciclovir, and septrin. She had a documented penicillin allergy.

**Fig 1:**
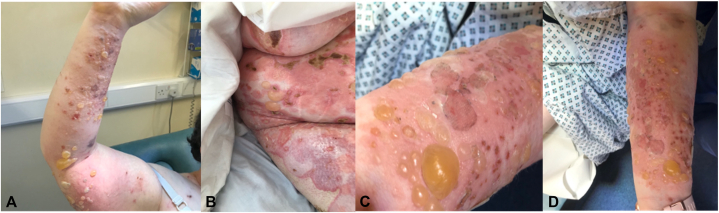
On presentation, prebullous rash progressed to tense bullae photographed on the upper limbs.

Three months after completing the course of nivolumab, she developed a pruritic, urticated, and eczematous rash predominantly affecting her trunk and proximal limbs. This rapidly evolved to become a widespread bullous eruption with tense intact bullae and erosions affecting her upper thighs, back, and arms (Fig 1).

**Fig 2:**
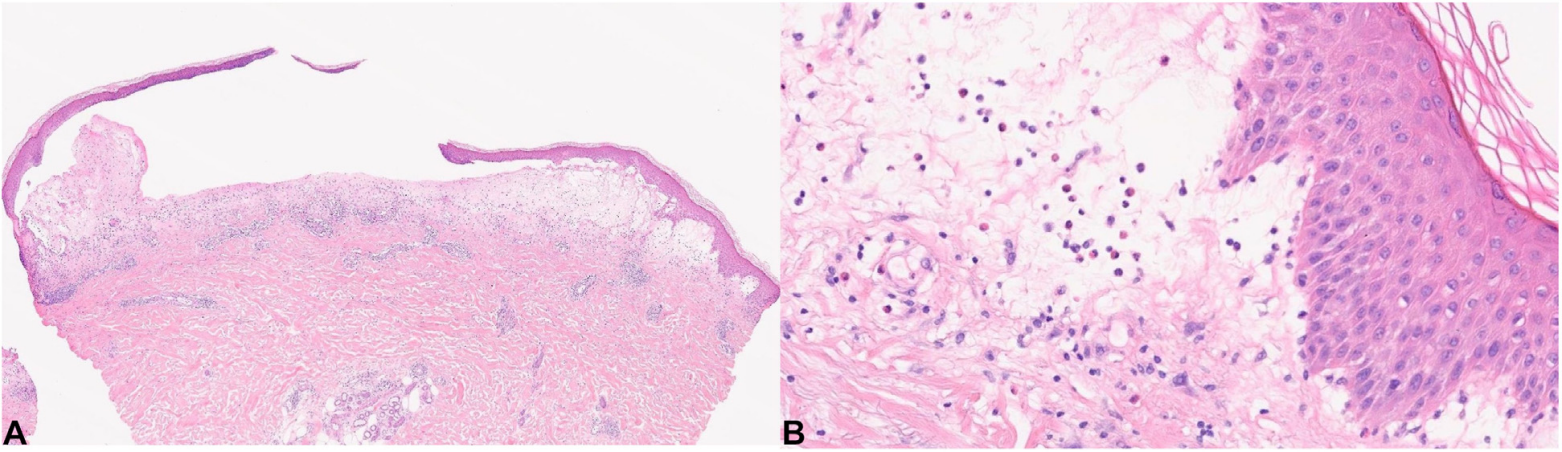
Histopathological findings **(A)** skin punch biopsy from the left forearm shows a subepidermal bulla containing numerous eosinophils, with intact and normal overlying epidermal roof (haematoxylin and eosin staining, original magnification ×2) and **(B)**, on higher power, the adjacent and underlying dermis is edematous and contains a marked perivascular chronic inflammatory infiltrate with abundant eosinophils (haematoxylin and eosin staining, original magnification ×20). These histologic features are consistent with the clinical impression of BP. Direct immunofluorescence findings confirmed the diagnosis.

A skin biopsy taken at this time demonstrated subepidermal bullous formation with abundant eosinophils (Fig 2). Direct immunofluorescence showed linear IgG and C3 along the dermoepidermal junction. Enzyme-linked immunosorbent assay tests demonstrated positive serum antibodies against BP-180 with an antibody titer of 193 U/mL and BP-230 with an antibody titer of 90 U/mL. These findings were consistent with a diagnosis of BP.

**Fig 3:**
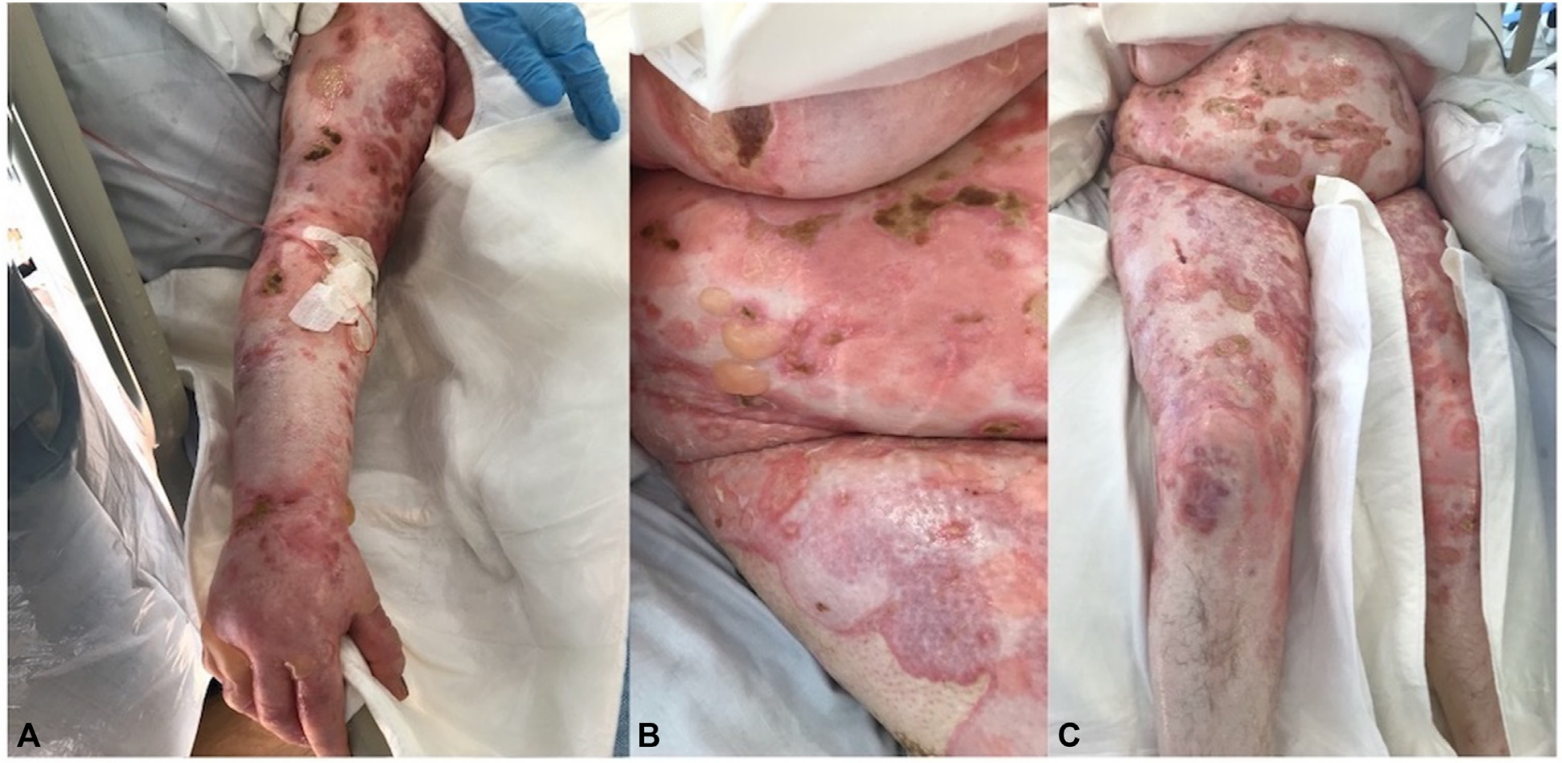
**A-C,** Clinical photographs demonstrating widespread bullous eruption with bullae of varying evolution and erosions.

The patient was initially treated with oral prednisolone 0.5 mg/kg, doxycycline 100 mg twice daily, and topical clobetasol propionate. Mycophenolate mofetil 1 g twice daily was added as a steroid sparing agent; however, this resulted in cytopenia. Her skin continued to blister despite treatment necessitating admission as an inpatient (Fig 3). At this point, the bullae and erosions involved over 70% of her body surface area with painful crusted erosions on her back, sacrum, and breasts. She required treatment with IVIg 2 g/kg, which was given over 3 days. However, despite these measures, she continued to deteriorate rapidly. In light of her severe and refractory BP, she was prescribed IV rituximab 1 g 1 week later. During this time, our patient required intesive care unit admission and courses of IV antibiotics to manage recurrent bacteremia, candidemia, and septicemia.

Despite the aforementioned interventions, the patient then required transfer to a tertiary referral center for plasma exchange. She completed 6 cycles of plasma exchange under joint hematology and dermatology care. There was significant clinical improvement following plasma exchange with less blistering. She remained on high-dose oral prednisolone 80 mg once daily but relapsed when the oral prednisolone was tapered.

In an attempt to begin to reduce oral steroids and gain disease control, omalizumab 300 mg was added due to further blistering and an elevated serum IgE measuring 348 kU/L (<114). This resulted in significant clinical improvement within 2 weeks of commencing treatment. Her skin improved and stabilized sufficiently to allow for discharge home after 5 months in the hospital. Unfortunately, over the following months, the patient was readmitted to the hospital because of worsening blistering, pain, and itch. The dose of prednisolone was increased again to 40 mg daily and dupilumab 300 mg alternate weeks was added in combination with omalizumab. A decrease in itch was noted within days of commencing dupilumab and her skin improved allowing discharge from the hospital.

**Fig 4:**
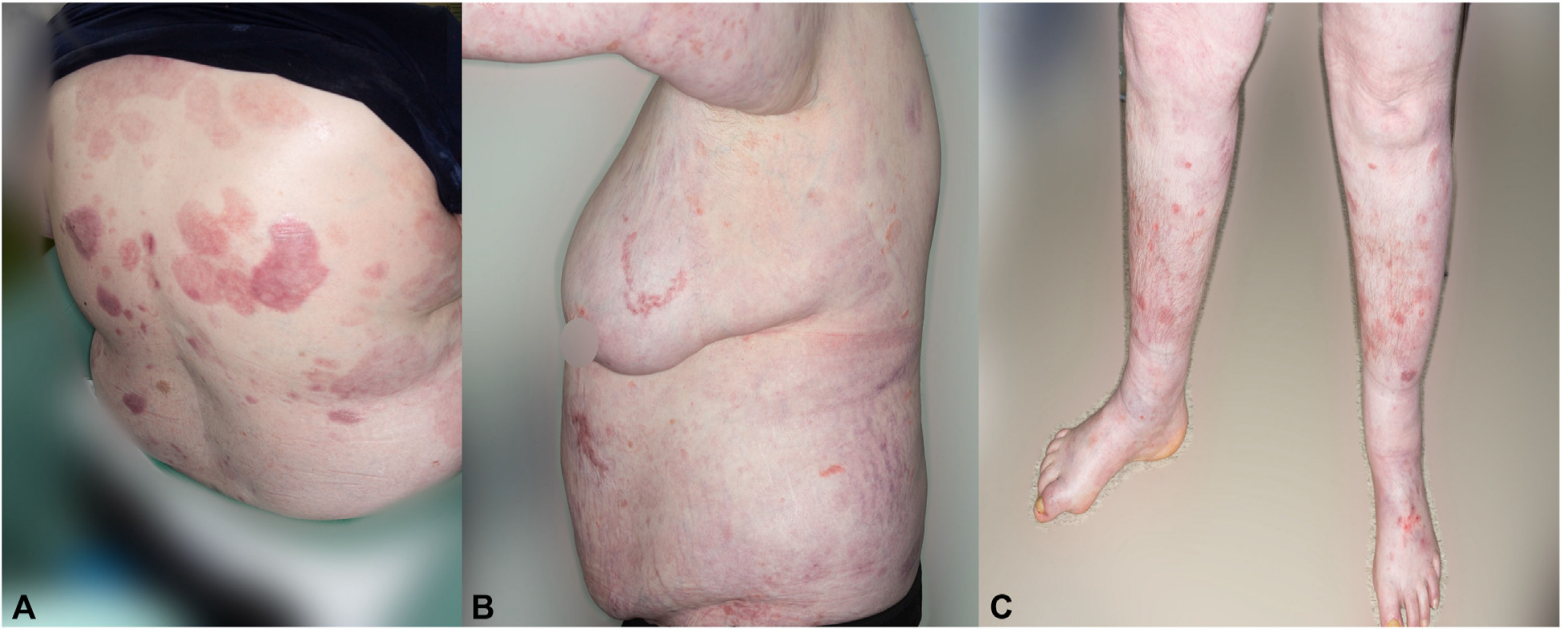
**A-C,** Clinical photographs of patient while on dupilumab and omalizumab in combination. Blistering has stopped and postinflammatory pigmentation is seen.

This combination therapy allowed us to slowly taper the patient off all oral steroids with the patient remaining blister-free. Serum enzyme-linked immunosorbent assay demonstrated anti-BP-180 reduction from 193 U/mL before treatment with omalizumab and dupilumab to 81 U/mL 6 months later. An attempt to stop omalizumab 6 months later resulted in a prebullous eruption on her forearms, which confirmed its effect. We have now been able to cease omalizumab and doxycycline and the patient remains blister free on dupilumab monotherapy 2 years later (Fig 4).

## Discussion

BP secondary to immunotherapy has presented a new cohort of patients with many unique therapeutic challenges. A greater understanding of the pathophysiology of BP has led to the off-license use of newer targeted biologic therapies. Dupilumab and omalizumab are emerging as potential targeted treatment options, particularly in cases recalcitrant to standard treatments. To our knowledge, the first case report of BP treated successfully with dupilumab dates back to 2018.^5^ Since then, further case reports have demonstrated its efficacy, not only resulting in significant clinical improvement and blister reduction but also in itch reduction. The favorable side effect profile of dupilumab makes it an attractive treatment option for elderly and frail patients, as well as those with underlying malignancies. In 2021, Klepper and Robinson^6^ reported a case of nivolumab-induced BP successfully treated with dupilumab. The successful use of omalizumab in BP was reported much earlier by Fairley et al^7^ in 2009. Since then, further case reports have demonstrated the efficacy of omalizumab, with inhibition of new bullae, resulting in less pruritus, and a decrease in eosinophil count.^4^ Seyed Jafari et al^8^ reported the first case of omalizumab and dupilumab being used in combination to control severe treatment-refractory BP. To our knowledge, LIBERTY-BP ADEPT study is the first randomized, controlled trial designed to evaluate the efficacy and safety of dupilumab in patients with moderate-to-severe BP.^9^

## Conflicts of interest

None disclosed.
